# Novel deletion alleles of a *C. elegans* gene Y73E7A.1, named as *tm6429* and *tm6475*

**DOI:** 10.17912/W2808M

**Published:** 2017-10-03

**Authors:** Yuji Suehiro, Sawako Yoshina, Sayaka Hori, Shohei Mitani

**Affiliations:** 1 Department of physiology, School of Medicine, Tokyo Women’s Medical University, Shinjuku-ku, Tokyo, 162-8666, Japan

**Figure 1.**
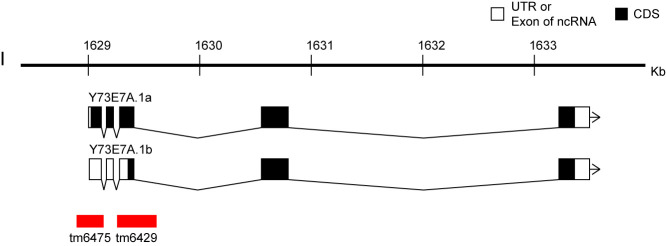


## Description

We report *tm6429* and *tm6475* as novel deletion alleles of the gene *Y73E7A.1* that is a homologue of mammalian Coiled-coil domain containing 124 (Ccdc124)1. The Ccdc124 is a conserved gene from invertebrates to human. In human cell lines, Ccdc124 is a component of the centrosome during interphase and at the G2/M transition. During cell division, Ccdc124 relocates to the midbody at telophase and acts as an essential molecular component in cytokinesis2. The alleles were isolated from the comprehensive screening of gene deletions generated by TMP/UV3. In the screening, both the alleles were detected by nested PCR using the following primer sets, 5’-GTGTGAATCGAGGAGGCGCA-3’ and 5’-TTTCCAGTCCGGCAGGCGAT-3’ for first round PCR and 5’- AACGGCAAACGCGCTCTATG-3’ and 5’- CGTGTGCACGTGGAAGTCCA-3’ for second round PCR. By Sanger sequencing, the 30bp flanking sequences of the alleles *tm6429* and *tm6475* were identified as TTTTAAATCGATTTTTGAGCACCAAAATTA- [355 bp deletion + 1 bp insertion (T)] – TTAAAAATGAGAAAAAATGGGGAAAAAATT and CAAACGCGCTCTATGGAGAATGTGGAATTA- [242 bp deletion] – TTTTATATAGGATTTTAATTTTCAGGCCAC, respectively. Based on the information about the splicing isoforms of *Y73E7A.1* (WormBase, http://www.wormbase.org, WS259), the start codon of *Y73E7A.1a* and *Y73E7A.1b* transcripts are deleted in *tm6429* and *tm6475*, respectively (Fig. 1), suggesting that those alleles may be usable for the analysis of isoform specific function. ​

## Reagents

FX06429 *Y73E7A.1* *(**tm6429**)* I (Not outcrossed) FX06475 *Y73E7A.1* *(**tm6475**)* I (Not outcrossed)
